# Discussion on Anæsthetics in the Therapeutic Section of the British Medical Association, at the Annual General Meeting at Bournemouth, 1891

**Published:** 1891-07

**Authors:** 


ARTICLE II.

### DISCUSSION ON ANÆSTHETICS IN THE THERAPEUTIC SECTION OF THE BRITISH MEDICAL ASSOCIATION, AT THE ANNUAL GENERAL MEETING AT BOURNEMOUTH, 1891.

The President of the Section (Dr. Snow, of Bournemouth), called upon Dr. Lauder Brunton, F. R. S., of London, who narrated the share he had had in the experiments undertaken by the Hyderabad Chloroform Commission. The origin of the Commission was the munificence of His Highness the Rajah of Hyderabad, who at the instigation of Surgeon-Major Lawrie, B. M. Edin., has given a large sum of money to defray all the expenses incident to the undertaking. Dr. Lauder Brunton stated that the conclusions at which the Commission had arrived were the result of some six hundred experiments, which, although few in number, yet represented a much larger number, since each one was a death. He projected upon a screen photographs of the actual tracings obtained and explained in detail the mode of experiment. Thus, when a tracing was taken, *e.g.*, of blood pressure, the record was made upon two revolving cylinders, the one a slow, the other a quick movement, which enabled the observer to record, not only the main curves, but also the details. In this way, blood pressure tracings were demonstrated, which revealed the fact that under chloroform there was a constant fall, which, however, never reached a dangerous point unless the respiration of the animal was interfered with. When this occurred there was at once a marked fall of blood pressure, which resulted in death unless the obstruction to breathing were removed. The experience of the Commission led them to believe that, provided this interference with respiration was guarded against, chloroform gave rise to no dangerous symptoms. The fall of blood pressure was regarded as a protection, the result of vagal action being to slow the heart, and so lessen the rate of circulation of the chloroform-laden blood stream.

Dr. Lauder Brunton, referring to the assertion of Professor Wood, of Philadelphia, that he had found heart failure to occur as a result of the fall of blood pressure, even when the respiration continued, stated that the Hyderabad Commission had made a number of tracings similar to the one upon which Professor Wood based his conclusion, and had at first been unable to account for them, but investigation had shown that the true cause of the tracing was the formation of a clot in the canula, and that when this was removed the heart trace was restored. Dr. Lauder Brunton said that the Commission expected to find the "fatty heart" play an important part in the matter, but their experiments made upon animals which were poisoned by phosphorus, and then subjected to chloroform had not shown that these creatures died from heart failure. In conclusion, Dr. Lauder Brunton emphasized a statement which he had made in opening his address, namely, that death under, or during the administration of chloroform must be carefully distinguished from deaths due to chloroform poisoning, as deaths from shock had occurred in pre-chloroform days, and took place now in cases when the patient was not thoroughly under chloroform at the time of the operation.

Dr. Shore (Cambridge) gave an account of some experiments conducted by Dr. Gaskill and himself, which were undertaken to investigate the cause of the fall of blood pressure which occurs in chloroform administration. The experiments were made on animals in which tracings of the respiration and blood pressure were taken. The chloroform was given in various ways. (1.) Inhaled through the trachea. (2.) Introduced into the carotid or vertebral artery, either by injection of a drop directly into the blood stream, or by transfusion of blood from the arteries of another animal to which the chloroform was given. (3.) Injected into the jugular vein. (4.) Applied directly to the 4th ventricle of the brain. The experiments show that chloroform acts powerfully on the respiratory centre in the medulla, leading to a slowing and an arrest of respiration. Less than one minim of chloroform injected into the vertebral artery is sufficient to stop the respiration at once. The neighboring vasomoter centre in the medulla is not paralysed, but, on the contrary, stimulated by the chloroform, and a rise of pressure results. Chloroform introduced into the jugular vein leads to a weakening of the heart's beat and a fall of blood pressure, but if the injection is continued, the respiration ceases before the heart absolutely stops. Anæsthesia in animals may be produced by chloroform with little or no fall of blood pressure if chloroform be administered with plenty of air. A fall of pressure, however, usually takes place, and this is due to direct action of chlorof rm on the heart, and not to a paralysis of the vasomotor centre. The respiration in all cases ceased before the heart beat, but in some cases the blood pressure, in consequence of the weakened heart, was obviously insufficient,and death must be ascribed in these cases to failure of the circulation.

Dr. Dudley Buxton (London) then opened the discussion from the clinical point of view. He said, he could not but preface his remarks by a few words expressive of the deep regret which he and all present must feel that Dr. Charles Sheppard, whose name appeared upon the programme, had since its publication passed away. Speaking in warmly eulogistic terms ot Dr. Sheppard, he commenced the discussion by saying that there were four sources at least of information derivable from clinical sources about anæsthetics and their behavior. These were (1) published accounts of deaths occurring under an anæsthetic; (2) accounts of dangerous and anxious cases under anæsthetics; (3) the results of necropsies made upon persons dying under an anæsthetic; and lastly (4) the results of experiments made upon human beings who had placed themselves voluntarily under experiment. The results of statistical enquiry showed facts which proved that deaths occurred both among the apparently healthy and robust as well as those who were diseased. It was said that fatalities of the strong and robust were deaths under fright rather than from chloroform, but this explanation was insufficient, since similar occurrences should, if this were the case, take place in a like proportion when ether, laughing gas or other anæsthetic was given, and no such deaths were recorded.

Again, the so-called deaths from fright were so exceedingly rare that it could scarcely be accepted that such an explanation accounted for more than a small fraction of the cases. Of the residuum of cases, most occurred in the initial stage of chloroformization and were characterized by the most terrible suddenness of onset of the symptoms of danger. They occurred in persons who had taken chloroform previously and both among those who were fearful and those who were free from timidity. In this place he must point out that persons who expressed great fear of the anæsthetic and who thought they would die under it, commonly, in his experience, took the anæsthetic as well as other persons, so that fear was by no means a constant factor in sudden death under chloroform. Then again, these sudden deaths were very often in cases when a trifling operation was to be performed, the reduction of a dislocation being frequently the occasion when death occurred. Death often took place before any surgical measures were commenced and both when the chloroform was being given by experts or by tyros. In all these deaths the heart was said to have stopped either simultaneously or before respiration. Among the diseased Dr. Dudley Buxton thought the fatty generation of the heart was at all events statistically the most common pathological lesion associated with sudden death from chloroform. He was aware that in this particular clinical results were at variance with those of the Hyderabad Commission, but he was not there to criticise the Commission's work, although he thought it was open to severe criticism upon several of its findings, he would therefore only say that he was sure that the results obtained in the lower animals by poisoning them with phosphorus were very different from those occuring in individuals whose heart muscle was the seat of fatty change. In this last case, the fatty heart was but one lesion of many, all of which were expressions of a general state of malnutrition and enfeeblement. Statistics again showed the fallacy of the once universally accepted statement that women and children were less liable to death from chloroform than men. Snow, whose book Dr. Dudley Buxton regarded as still a model of scientific research and method of dealing with the subject under discussion, had been led into error by the comparatively small number of cases he had been able to collect. Young children, old persons, and parturients had all died from chloroform. The supposed immunity of children was due to the less frequency of their requiring chloroform as well as the usually more healthy state of their body. Old persons having surmounted the dangers of life were also presumably more vigorous, and so better able to escape the depression brought about by the drug. Women in childbed usually took chloroform under the most favorable conditions, seldom inhaling enough to carry them beyond the point of analgesia, and always with an extremely dilute vapor. Dr. Dudley Buxton then gave examples of the second source of information, viz.: cases in which no death but only dangerous symptoms had supervened, and urged the immense importance of the investigation of the cases. Referring to ether, he said that he believed deaths from this agent were in the very largest proportion of cases preventable. As a matter of fact, few persons understood the proper way to give ether, and so struggling and distress arose, and it was these which gave ether a bad name and jeopardized lives. He urged the necessity for careful and thorough training of young doctors before they were entrusted with the responsible duties of an anæsthetist. He entered a vigorous protest against the statement of Dr. Lauder Brunton, that ether did not produce anæsthesia unless the patient was concurrently semi-asphyxiated. Dr. Dudley Buxton then detailed various interesting points concerning the symptoms brought about by chloroform and ether when given to patients in different postures, and for various operations, mentioning empyemas as cases of peculiar danger and difficulty, and stated that he had adopted the plan of rectal etherisation for them.

Mr. Pridgin Teale (Leeds) gave a forcible paper in favor of ether. He had collected the published cases of death under anæsthetics for the past year, and had found that chloroform was the agent which killed It was startling to learn that in spite of the vaunted safety of chloroform it had been the means of killing so large a proportion of patients, and what was more distressing, they had died when operations of the most trivial kind were being performed. He had worked with chloroform for many years but had been induced to try ether, and the increase in his feeling of comfort and security since then was remarkable. Ether he regarded as a safe anæsthetic, which, if properly given, need give no distress, and possessed the great advantage that when the patient was once under its influence but little more of it was required. Mr. Pridgin Teale then described in detail the method he adopted, which was that commonly followed when Clover's small portable regulating inhaler is used. He laid considerable stress upon the importance of attention to details, *e. g.*, having a large bag into which the patient breathes, &c. In the case of children he commonly deadened sensibility by giving a few whiffs of chloroform, and following up with the ether.

Mr. George Eastes, M. B. (London), next read a communication in which he proceeded to criticise adversely and at some length the conclusion at which the Hyderabad Commission had arrived. He considered it unsafe to argue wholly from the lower animals to man, and especially when results conflicted. The difference of climate between India and Europe probably also played an important part in the mattar. But even accepting the experiments upon lower animals as tantamount to those on man, he reminded his hearers that the conclusions were at variance with the results attained by other observers. Personally he attached more importance to the clinical aspects of the matter, for after all each administration of an anæsthetic to a patient really amounted to an experiment, and the experience of many thousands of such cases went dead against the researches of the Hyderabad Commission. It must be remembered that the conclusion of the Commission had now been published some time, and no doubt most of those who administered chloroform would be aware of them, and very many would follow out the directions to the letter, yet in spite of this the death rate was, if anything, higher than before, and fatality after fatality was recorded in the journals. Surgeon Major Lawrie could boast clinically of a most remarkable record of cases in which chloroform had been safely administered, but the difference of the environment of his patients from that of Europeans possibly account for that. Indians were accustomed to a different dietary, the race traits rendered comparison between them and European patients inadmissible. Anæsthetization was in every case an approach to death, the various agents used were different only in the margin they left between sleep and dissolution, and chloroform gave less margin than ether. This was what clinical evidence showed. Mr. Eastes then described the methods which he thought were best.

The Secretary of the Section (Dr. Childs, of Weymouth), after speaking of the great importance of clinical investigation, suggested that a Committee should be appointed with the object of investigating various questions concerning anæsthetics, such as the relative safety of chloroform and ether, the best methods of administration, and so on.

This having been embodied in a resolution, it was put to the meeting and carried, Dr. Lauder Brunton, Dr. Dudley Buxton, Mr. Pridgin Teale, and the officers of the Section (*ex officio*) being requested to serve upon it.

Dr. Sidney Coupland (London) warmly commended the suggestion of the formation of the Committee, as he believed the work it proposed to carry out would prove most useful. He commented upon the anxiety which physicians often felt about their cases, *e.g.*, those of empyema, when an anæsthetic was to be administered. He had a case under his charge which, when transferred to a surgeon for surgical treatment, had died under the anæsthetic. Practical rules for their guidance, in advising or discountenancing the use of one or other anæsthetic in such cases, would prove of very great value to physicians.

Dr. Copeman said that he had been compelled to give chloroform to a large number of the lower animals, and in his experience whenever he used methylated chloroform the animal died, but not when the purer forms were employed. He had been assured by manufacturers that the methylated chloroform was to all intents and purposes as pure as any, but this was at variance with the behavior of his animals. So sure was he that when by any accident the methylated chloroform was used, the symptoms produced would always show him the error. He also thought an important point was to warm the air which passed through the chloroform: since adopting this precaution, his results with the lower animals were better.

Mr. Harsant (Bristol) thought statistics were of little value by themselves, for the results of different people under varying conditions were most dissimilar. In support of this, he read a number of statistics of deaths under anæsthetics, which were certainly very conflicting. With regard to the mode in which chloroform killed, it was disappointing to find scientists so much at variance. Dr. Lauder Brunton, Professor Wood and Dr. Gaskill, seemed to almost contradict each other's results. Personally, he had no doubt that chloroform killed first by causing primary heart failure, and secondly, by its action upon the heart centre in the medulla. He had used chloroform about as frequently as ether, but had never met with the same dangers and difficulties with the latter as with the former agent. He narrated the only case of death in his practice when ether had been given, but as the patient had regained consciousness for some while, and death had occurred suddenly upon his sitting up in bed, he attributed it to syncope and not to ether.

Mr. Neve (India) spoke of ten years's experience in hospital practice in India, during which he had seen chloroform administered 4,000 times without a death. These cases were in different parts of India, the range of temperature being very wide. He believed many deaths were the result of an insufficient dose of the chloroform, and he narrated a case illustrative of this. He always followed the Edinburgh method.

Dr. Horsfall, a local practitioner, expressed his personal belief in the safety of chloroform when it was given pure and given properly.

Dr. Renton (Glasgow) had learned how to give chloroform under Syme and to believe in it. He had met with several anxious cases and not a few difficulties. He had since been induced to employ ether and had now given up chloroform as he found with ether there was less anxiety, and whatever difficulties or dangers arose were easily met and overcome, which was not the case with chloroform. There was but one desideratum, and that was a simpler apparatus: Clover's smaller inhaler, admirable as it was, was yet more complex than many men cared to employ.

Dr. Tyson remarked that one most important matter was that even if ether and chloroform could be shown to be equally dangerous, yet dangers arising from ether were so much more gradual in their onset, and so much more easily grappled with, that there would always be a much more favorable record for ether.

The Hon. Dr. Lefevre (Australia) thought that a colonial's experience might be of interest. There was a growing feeling in favor of ether, but the methods employed were as yet far from satisfactory.

Dr. Lauder Brunton replied,—and

Dr. Dudley Buxton, after replying, gave, at the request of several persons present, a brief account of the methods he employed in practice.

The Session closed with some appropriate remarks of the President, who said he had never been present at a more eloquent discussion, nor one in which the disputants, although holding very opposed views, yet had avoided any language which could give rise to unpleasantness or misunderstanding. —*London Dental Record.*

